# Inferring microRNA regulation: A proteome perspective

**DOI:** 10.3389/fmolb.2022.916639

**Published:** 2022-09-08

**Authors:** Dan Ofer, Michal Linial

**Affiliations:** Department of Biological Chemistry, Institute of Life Sciences, The Hebrew University of Jerusalem, Jerusalem, Israel

**Keywords:** Automated machine learning, miRNA target interactions, AI classifier model, GPCR, miRTarBase, paralogs, post transcriptional regulation, protein family

## Abstract

Post-transcriptional regulation in multicellular organisms is mediated by microRNAs. However, the principles that determine if a gene is regulated by miRNAs are poorly understood. Previous works focused mostly on miRNA seed matches and other features of the 3′-UTR of transcripts. These common approaches rely on knowledge of the miRNA families, and computational approaches still yield poor, inconsistent results, with many false positives. In this work, we present a different paradigm for predicting miRNA-regulated genes based on the encoded proteins. In a novel, automated machine learning framework, we use sequence as well as diverse functional annotations to train models on multiple organisms using experimentally validated data. We present insights from tens of millions of features extracted and ranked from different modalities. We show high predictive performance per organism and in generalization across species. We provide a list of novel predictions including *Danio rerio* (zebrafish) and *Arabidopsis thaliana* (mouse-ear cress). We compare genomic models and observe that our protein model outperforms, whereas a unified model improves on both. While most membranous and disease related proteins are regulated by miRNAs, the G-protein coupled receptor (GPCR) family is an exception, being mostly unregulated by miRNAs. We further show that the evolutionary conservation among paralogs does not imply any coherence in miRNA regulation. We conclude that duplicated paralogous genes that often changed their function, also diverse in their tendency to be miRNA regulated. We conclude that protein function is informative across species in predicting post-transcriptional miRNA regulation in living cells.

## Introduction

MicroRNAs (miRNAs) post-transcriptionally regulate genes across all animals and plants. miRNAs are a class of short (∼22 nucleotide) noncoding RNAs (ncRNAs). Mature miRNAs act *via* complementarity with their target mRNAs. This pairing takes place mostly at the 3′-UTR of the transcripts (Romero-Cordoba et al., 2014). In mammals, such binding leads to translational repression of the target and direct or indirect degradation of the miRNA-targeted transcript *via* deadenylation and decapping of its target (Valencia-Sanchez et al., 2006; O'Brien et al., 2018). miRNAs play key roles in a broad range of cellular processes and the response to changes in the environment (Leung and Sharp, 2010). The miRNA profile is tissue-specific and an indicator of cell identity (Mahlab-Aviv et al., 2021). Their ability to maintain cell and tissue homeostasis is critical, with many miRNA genes implicated in human diseases such as metabolic, inflammatory, and neurodegenerative diseases (Vishnoi and Rani, 2017). In cancer samples, the miRNA composition changes along with the tumorigenic process. Therefore, the miRNA profile carries useful diagnostic and prognostic potential for tumor typing and patient survival.

With the maturation of deep sequencing methodologies for small RNA identification, the number of reported mature miRNAs has drastically increased. The exhaustive catalog of miRNAs (miRBase v. 22) (Kozomara et al., 2019) reports on 1917 genes that account for 2625 mature miRNAs from humans, and 1,234 and 1978 genes and mature miRNAs from mice, respectively (Quillet et al., 2019). With a set of strict criteria imposed by miRBase, only a quarter of the listed miRNAs from humans are labeled with high confidence. Many of the rest have yet to be experimentally confirmed (Alles et al., 2019). From the standpoint of miRNA targets, it has been demonstrated that many human genes are under selective pressure to maintain miRNA pairings (Friedman et al., 2009). Despite an increase in the number of validated miRNAs, the estimated number of regulated genes remained between 60 and 80% of all human protein-coding genes (Sayed and Abdellatif, 2011; Huang et al., 2019).

In the last 15 years, computational miRNA-target prediction algorithms and tools have been developed (Sethupathy et al., 2006; Yue et al., 2009; Riffo-Campos et al., 2016). Almost all of these predicting tools are based on features derived solely from the genomic sequence. Major features include seed complementary, evolution conservation, free energy, CG content, the relative position of miRNA binding sites (MBS) at the 3′-UTR, and more (Ritchie et al., 2009). Most tools suffer from a large number of false positives, poor accuracy and sensitivity, and show a great degree of inconsistency among them (Min and Yoon, 2010).

High throughput methodologies (e.g., CLIP-seq, CLASH, CLEAR-seq) were used to conduct hundreds of experiments to infer miRNA-mRNA interactions (Li et al., 2014; Karagkouni et al., 2018). These experimental methods allowed us to assess the reliability of the different miRNA-mRNA prediction models. In general, the match between the experimental results and the computational predicting methods is poor. Experimental observations (e.g., CLIP data) and sequence-derived information about miRNAs and mRNAs are used to determine whether a specific transcript is a genuine target of miRNAs.

In this study, we address the question of whether a gene is a target of direct regulation by any miRNA based on their protein products, using a supervised machine learning approach. The goal is to predict if a protein is subject to direct regulation by “any” miRNA at a central probability. The underlying notion is that the coding regions of most genes are under strong negative selection forces and potentially include information that determines the essentiality of a gene under miRNA regulation, irrespectively of a specific combination of miRNAs. We use miRTarBase 2020 (Huang et al., 2020) as an experimentally validated ground truth dataset. We trained the system using experimentally validated resources for human, mouse, and other model organisms and reached high performance on the task of predicting gene-miRNA interaction, using primarily protein and minimal sequence level attributes. We included multimodal inputs from proteins and generalized across different species. We also evaluated genomic information and compared it to the proteomic model. We present an in-depth analysis of both novel and established features, extracted automatically using an AI-assisted machine learning framework (SparkBeyond). We present a list of candidate miRNA gene predictions from less studied organisms. Our model highlights the value of information embedded in the functional proteome in revealing the complexity of regulation by miRNAs.

## Methods

### Database: miRNA-target interactions

We used miRTarBase 2020 (V9) as a gold standard for miRNA-Target interactions (MTIs) (Huang et al., 2020). miRTarBase compiled experimentally validated MTIs, mostly from mice and humans. The entire database collected 4.5 M data points, based on CLIP-seq experiments, as evidence for human MTIs (covers ∼3000 miRNAs and 17,400 genes), and 0.7 M mouse MTIs (covers 2250 miRNAs and 14,300 genes). It is used as a ground truth for training. The dataset was downloaded from miRTarBase 2000 (Huang et al., 2020). The experimental results in miRTarBase 2020 (V9) have been associated “weak” or “strong” evidence. Weak support refers to data collected from high-throughput experiments (e.g., CLIP-based NGS experiments, pSILAC proteomics), while strong evidence is compiled from targeted experiments such as quantitative RT-PCR (qRT-PCR), Western blots, and reporter assays. We defined a target as positive (i.e., miRNA regulated) by having “strong” experimental evidence or at least two unique “weak evidence” experiments. The remaining genes from miRTarBase with a single “weak” experimental evidence were labeled as “likely positives” (0). These were treated as positives for the purposes of downstream analyses, unless otherwise stated. We note that excluding these “weakly labeled” samples improved modeling performance across all organisms (not shown). All other genes were marked as “negatives”, i.e., not targeted by the specific miRNAs (“-1”).

### Database: Proteome

Proteins were downloaded from UniProtKB for all organisms analyzed in this study. We used curated and manually reviewed SwissProt proteins (Breuza et al., 2016), except for organisms that are limited in the protein annotations where all full-length proteins were used (excluding fragmented sequences). Proteins annotated with no experimental evidence for their existence by UniProtKB-SwissProt were excluded, as by definition there could be no experimental evidence for their miRNA regulation. Altogether 45,846 proteins were analyzed. We also analyzed the proteomes of *Danio rerio* (zebrafish) and *Arabidopsis thaliana* (mouse-ear cress).

We identify genes with their matched proteins. Proteins from UniProtKB were mapped to the genes listed in miRTarBase, TargetScan and TreeFam according to their primary gene name. To connect genomics with protein identifiers, we mapped human genes by their primary gene names. To avoid inaccuracies, we applied a strict rather than fuzzy mapping, and uniquely mapped 76% of the human proteins across these different resources. A negligible number of proteins with no primary gene name were removed.

### Extracted features

A wide range of metadata about each protein from UniProtKB was used as proteins’ features. These included the proteins’ amino acid sequence (e.g., amino acid composition, counts, n-grams), molecular weight, protein length, functional keywords (e.g., secreted, membranous), gene ontology (GO) annotations for all three branches: molecular function, cellular component, and biological process), pharmaceutical uses, tissue specificity, protein family, post-translational modification, involvement in disease, compositional bias, non-terminal residues, and more. In addition to the information associated with the proteins by UniProtKB, we also derived engineered features based on the primary features. These quantitative features include amino acid composition and k-mers, n-grams (e.g., combinations of keywords), and counts of known annotations (Ofer and Linial, 2015).

As a separated set, we extracted limited genomic data for the human proteome using the Biomart querying system of Ensembl (Yates et al., 2016). The derived features included: The length of the UTR (5′ and 3′), the counts of alternative splicing, chromosomal position, nucleotide counts, k-mers and their frequency, k-mer features extracted from the 3′-UTR genomic sequence, and transcript length.

### AutoML and feature extraction

Feature extraction, engineering, selection (Ofer et al., 2021), and ML model selection, parameter tuning, and training were performed using the SparkBeyond autoML framework (See patent/US20170017900A1). Previous work has shown the benefit autoML models, in order to comprehensively and automatically find possible predictive signals in complex data, including in biology and healthcare (Cohen et al., 2021). The system automatically extracts and ranks a wide range of compositional features from training data. The system applies hyperparameter tuning and evaluation of machine learning models. In this study, the SparkBeyond framework was applied to genomic, proteomic, and annotation data. Across the different problem formulations, the system generated on average ∼22 million candidate features per organism, prior to selection. A maximum of 300 features are selected and used for the ML models, based on the training data. The models are then evaluated on a held-out test-set, or a new test data-set (As in Table 2). Features include textual features (n-grams, k-mers, tokenization), counts, aggregations (e.g., max, min, average, decile), interactions (e.g., length of a sequence divided by weight), missing value imputations, similarities and more.

We use RIG (Relative Information Gain) as a measure of feature importance. RIG refers to the information gain measured as a reduction in entropy produced from partitioning a set with attributes a and finding the optimal candidate that produces the highest value.

IG(T,a)=H(T)−H(T|a),IG(T,a)=H(T)−H(T|a)

Where *T* is a random variable, and H (*T*|*a*) is the entropy of *T* given the value of attribute *a.* It encapsulates both the uplift of a feature (the increase in a class’s likelihood, given a binary partition induced by the feature), and the support (the number of samples covered by the feature). A feature with a high RIG is expected to be relevant for any model, given that it will have good support and lift.

We report as final evaluation on a held-out test set, comprising 20% of the data. Note that the feature extraction, selection and model evaluation and tuning is performed only against a subset of the training data, to avoid the risk of overfitting and model leakage. To improve interpretability, we limited the system to prefer “simple” features, at a slight cost to performance. Features are ranked by their marginalized, non-redundant mutual information score, as well as a custom regularization scheme to favor semantically simpler features (i.e., less composite functions). Performance in human only data was based on 20% held out stratified collection of 3810 test samples. The protein sequence only model uses just the statistics from the primary sequence [e.g., length, n-grams, amino acid composition (Ofer et al., 2021)], without any of the additional annotations or metadata.

We used standard definitions for the model’s performance, including precision and recall. In addition we report accuracy = (TP + TN)/(P + N), and the F1-score = 2 TP/(2 TP + FP + FN) using routine notations of T (true) and F (false), P (positive) and N (negative).

### Software

Data processing and analysis used the Python Pandas (McKinney, 2010), and Scikit-learn software packages (Raschka and Mirjalili, 2019).

Code, figures, processes and data are available at: https://github.com/LinialLab/microRNA-Protein-Regulation. Supplementary Table S1 provides links to a collection of analyses for each of the ML steps including pre-processing, features, predictions, model. The analyses allow an in-depth assessment of feature clustering, summary tables, and plots for visualizations). See reports in https://github.com/LinialLab/microRNA-Protein-Regulation/tree/main/reports/.

## Results

### Problem definition

In the context of cells and tissues, genes that respond to miRNA regulation comprise direct targets (i.e., miRNA binds to the 3′-UTR of a gene’s mRNA, affecting transcript stability and inhibiting translation) and indirect effects (e.g., miRNA that downregulates a transcription factor (TF), leading to attenuation of transcription of a set of TF-responsible genes). Another layer that is critical for assessing the regulation by miRNA concerns the combinatorial nature of the mapping (Balaga et al., 2012). In addition, quantitative competition on miRNA binding sites (MBS) results from a competition on the accessibility of the targets (Mahlab-Aviv et al., 2019). Such competition is governed by alterations in miRNA quantities and composition (Mahlab-Aviv et al., 2021), and the presence of circular RNAs (circRNAs), pseudogenes and long ncRNAs (lncRNAs) with MBS. This paradigm is referred to as competing endogenous RNAs (ceRNAs) (Denzler et al., 2014; Lai et al., 2016).

In this study, we question whether a coding gene is subject to direct regulation by any miRNA. Answering this question can lead to the design of in-depth experiments and computational screening. It can also lead to an accurate estimate of the outcome following cellular manipulation (e.g., by applying drugs). Moreover, generalization from miRNA-regulated genes across species contributes to the study of complex diseases in humans (e.g., Parkinson’s disease) in simpler organisms (e.g., zebrafish). This may lead to unveil disease mechanisms in a controlled setting. Manipulation of combinations of miRNAs is a valuable methodology for dysregulating biological pathways. To activate such an approach, one needs to know the genes that are likely to be subjected to miRNA regulation (Naamati et al., 2012). To answer whether a coding gene is subject to direct regulation by any miRNA, we considered features from a complete set of validated proteins within each of the studied organisms (coined “reviewed proteome” by UniProtKB-SwissProt). Our problem setting was defined as binary classification using supervised machine learning models. Scikit-learn’s linear logistic regression or random forest models were selected as the best models across the different runs by the SparkBeyond autoML framework (see Methods), outperforming other model architectures. Importantly, functional annotation provided by UniProtKB-SwissProt does not include miRNA-related knowledge, and potential target “leaks” were carefully excluded or filtered for (e.g., requiring direct evidence for a gene’s transcript).

We trained models on data and miRNA regulation annotations from different organisms. Supplementary Table S1 summarizes the data used for the studied organisms’ proteomes. We combine diverse protein functional annotations along with traditional sequence and biophysical features, as well as quantifying the relative contribution of universal genomic sequence-based features (i.e., not miRNA family specific). We identified key features that contribute to the models and suggest shared principles in miRNA regulation across species.

### Inconsistency in existing miRNA target predictions

Existing tools for predicting miRNA-gene interactions demonstrate poor consistency between tools and major resources. It is anticipated that it is mostly due to the very large number of false positives (Ding et al., 2010; Mackowiak, 2011). The question of what makes transcripts in any organism a good target could not be answered based on current tools (Min and Yoon, 2010). In an effort to reduce the flood of false positives, statistical framework across different predicting algorithms was developed, with the notion that miRNAs work together in a commutative fashion (Balaga et al., 2012; Friedman et al., 2014).

**FIGURE 1:**
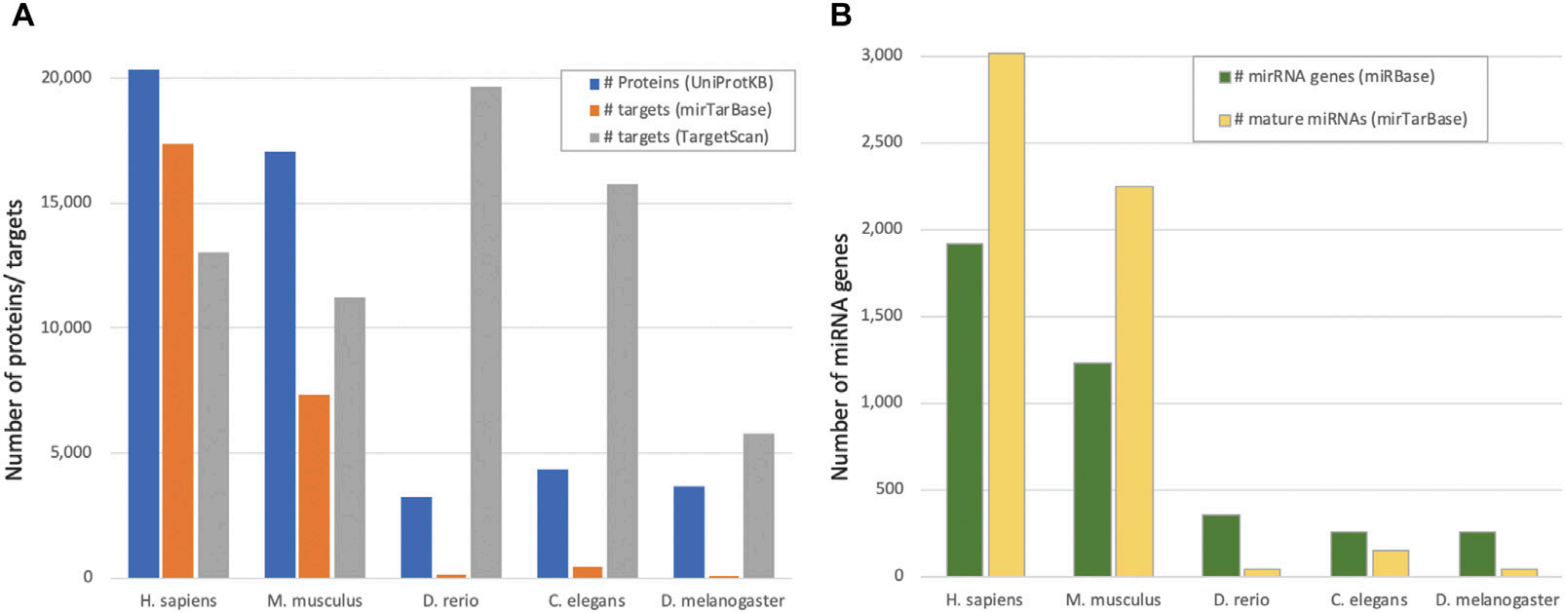
miRNAs and regulated genes by species. **(A)** Number of proteins from UniProtKB/SwissProt reported as “reviewed proteome” (see Methods) and the number of reported targets from mirTarBase as high confidence (by evolutionary conservation) reported by TargetScan, for five model organisms. **(B)**. The number of miRNA stem-loop genes (miRBase) and mature miRNAs (miRTarBase) for the model organisms as in **(A)**.

Experimentally validated targets (as derived from miRTarBase 2020) are expected to be of higher quality and consistency, and thus are used as the “ground truth” annotations. However, such annotations suffer from inherent biases. For example, it is likely that highly expressed transcripts will be detected more often than lowly expressed ones. Similarly, miRNAs that are expressed under defined conditions might be underrepresented experimentally. Obviously, some organisms are studied more than others (e.g., human and mouse), resulting in a biased view of how many miRNA targets there are in most organisms. Including data from experiments and evolutionary considerations were applied to overcome such limitations [e.g., (Gerlach et al., 2009)]. Nevertheless, the *Rattus norvegicus*, which is a commonly used model organism for researching human diseases and drugs, has twelve times fewer (validated) miRNA targeted genes relative to the mouse (3.1 vs. 37%). Figure 1A illustrates the disparity in the fraction of genes predicted to be regulated by miRNAs (as predicted by TargetScan) and those experimentally validated (by miRTarBase). We illustrate the TargetScan (release 8.0) (McGeary et al., 2019) as a reference point. It provides a score for each pair based on genomic and biochemical models of miRNA binding specificity. However, the algorithm was mostly tuned as a miRNA-mRNA predicting tool for mammals. The discrepancy in the proteomes of *D. rerio* (zebrafish) and *C. elegans* (worm) emphasizes the unproportionate number of predictions by TargetScan in view of the shortage of experimentally validated observations. Figure 1B shows that inconsistency is also detected in the number of confirmed miRNA genes. The proportion of confident miRNAs reported by miRBase reaches 70% of the entire miRNA gene list. In *D. melanogaster,* however, it is only 57% (out of a total of 258 genes reported in miRBase) (Supplementary Table S2). In humans, the fraction of confident miRNAs is only 26% (505 out of 1917 miRNA genes) and poor confidence miRNAs are those with minimal expression level or non-canonical stem-loop structure.

### Prediction of miRNA regulation across organisms

For each organism, we trained a model on 80% of its curated proteins (proteome filtered collection, see Methods) and presented results on the disjointed remaining 20% test set. Labels (miRNA regulation/non-regulation) were derived from experimentally validated miRNA regulation data (miRTarBase 2020). A few proteins in humans that lacked primary gene names were excluded in advance (a total of 66 out of the 18,874 proteins, leaving 18,808 valid proteins for the analyses). We identified 76% of human genes and only 37% of mice as regulated (validated) genes, with lower rates for other organisms.

Table 1 shows the performance of the protein models for different model organisms. Models were based on annotated protein data (coined “reviewed”). Model results are shown for the test set (which was not used in model training). We consider total instances as the number of genes after filtering and assigning a unique mapping across the different resources. The percentage of validated miRNA regulation refers to experimentally validated targets. The performance is presented according to the area under the receiver operating characteristic curve (rocAUC), precision, and recall (of the minority class) on the test set. We concluded that, despite the low coverage of annotated proteins in the fly and worm proteomes, the machine learning-based model successfully characterized genes that are apparently regulated by miRNAs. It also emphasized the commonalities and differences in miRNA regulation across model organisms.

**TABLE 1:** miRNA predictions per organisms.

Species proteins^a^	Precision (%)^b^	Recall (%)^b^	Total proteins	Validation as miRNA regulated (%)	rocAUC
*H. sapiens* (R)	63.4	27.8	18,808	76.0	76.9
*M. musculus* (R)	57.1	37	16,355	37.0	67.6
*R. norvegicus* (R)	33.3	4.3	7,519	3.1	87.4
*D. melanogaster* (R)	33.3	10	3,140	1.5	67.5
*D. rerio* (R)	0	0	2,799	1.0	79.0
*C. elegans* (R)	50	18.1	2,412	2.4	65.5
A. thaliana (R)	85.7	46.1	11,876	0.3	97.0
*D. rerio* (All)	72.7	40	24,730	0.7	93.6
*C. elegans* (All)	90.4	34.5	8,577	2.7	85.5
A. thaliana (All)	80	47.0	19,260	0.3	90.1

a Proteins extracted from UniProtKB-SwissProt. R indicates the reviewed and annotated protein set. The unified set including UniProt-TrEMBL, is marked as All.

b Precision, Recall values are for the predicted minority class, at default model threshold cutoff.

In mammals, it has been shown that most genes are directly regulated (based on CLIP experiments). However, the extent of miRNA regulation in invertebrate organisms remains unknown. Table 1 also tests whether a strict selection of the trained set of curated and well-annotated proteins (Reviewed, R) impacted the performance of the miRNA-regulatory predictor. We observed that for some of the tested organisms, the fraction of validated miRNA targets among the reviewed proteins is very small (e.g., 1% for *D. rerio*), which limits the ability to learn from this small intersection. In contrast, the performance of the model for *A. thaliana* which has a negligible number of experimentally validated miRNA-regulated genes is higher for the curated set relative to the full proteome (Table 1). We reran the models for *C. elegans*, and *D. rario* with the full-length proteome (marked All) and report on a substantial improvement in the rocAUC for models trained on the larger set.

### Generalizing between species

In addition to developing a species-centric model, we evaluated the ability of the models and features to generalize between species. We trained a model on all human proteins and evaluated it using all mouse proteins as a test set, and vice versa (mouse to human; Table 2). We observed an excellent stability of the results, with performance dropping only slightly compared to a dedicated model trained on the species’ own data. This supports our use of protein-based models to predict between different species, arguing that functional attributes generalize well between species. We show that the model trained on the reviewed proteomes or mouse led to improved performance for the zebrafish, fly and worm (Table 2). We concluded that training on high quality species allows reliable model generalization.

**TABLE 2:** miRNA predictions between organisms.

rocAUC score^a^	Trained on human	Trained on mouse
Evaluated on *H. sapiens* (R)	76.9	64.6
Evaluated on *M. musculus* (R)	75.0	67.6
Evaluated on *R. norvegicus* (R)	78	79.6
Evaluated on *D. melanogaster* (R)	73.4	77.9
Evaluated on *D. rerio* (R)	63.6	70.3
Evaluated on *D. rerio* (All)	76.2	68.5
Evaluated on *C. elegans* (R)	57.7	59.6
Evaluated on *C. elegans* (All)	51.0	60.4

a Proteins extracted from UniProtKB-SwissProt marked as reviewed and annotated protein set (R). In bold face are the higher performance for each species protein set, according to the training model.

### Protein features predict miRNA regulation

We observed a number of predictive attributes that were consistent across different iterations and even different organisms. We identified global attributes that are clearly in favour (or disfavour) of miRNA regulation. Features were ranked according to the calculated RIG (relative information gain) values, with higher RIG implying lower uncertainty for the target under the feature’s induced partition, i.e., greater information about it (see Methods). A full ranked list of features and their statistical properties is in Supplementary Table S1.

**FIGURE 2:**
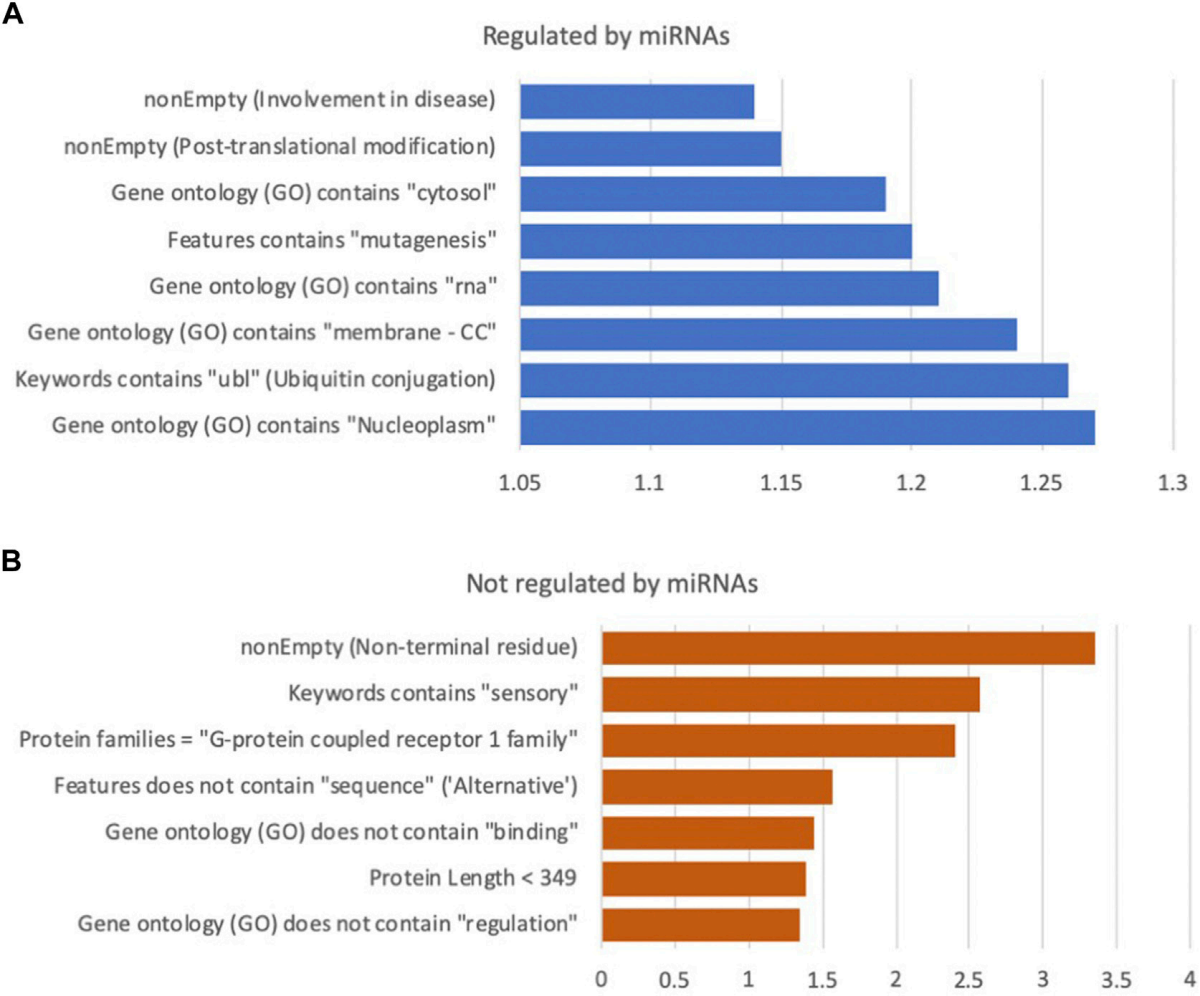
Selection of impactful features from the human protein model. **(A)** miRNA regulated, **(B)** not regulated by miRNA. The *x*-axis is the lift of selected features relative to the class prior baseline (76% regulated, 24% not regulated). All above features have an uncorrected *p*-value at least under 0.0001, using a Chi2 test on the test data. Full list of features and statistics in Supplementary Table S3, along with ranking by information gain (RIG). Note the different scale of the effect in **(A,B)**. CC: Cellular component branch of GO annotations.

**FIGURE 3:**
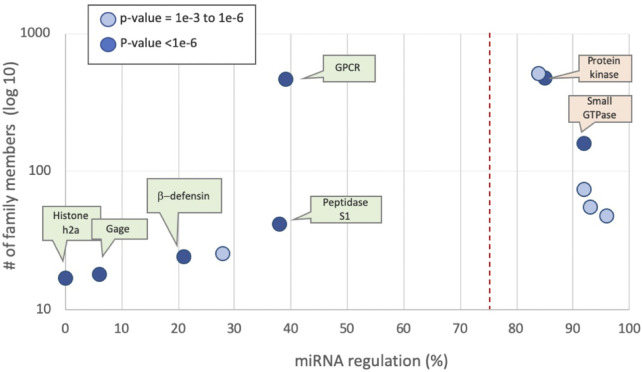
Protein families by mean percentage of miRNA regulation predictions of their members. Families defined by UniProtKB. Examples from human families are partitioned by the prior rate for miRNA regulation of 75% (dashed line). The *p*-value for each family member is marked by the two-sided binomial test. The most significant families (*p*-value <1E-6 are indicated) are listed, colored green and orange by their predicted values of 0 and 1, respectively. Supplementary Table S4 lists protein families along with their prediction statistics.

A high RIG value encapsulates a high degree of confidence with strong statistical significance for the discrimination power (chi2 *p*-value <1.0E-04 on the test set). Figure 2 shows the partition of the top 15 features with high uplift in discriminating for miRNA regulation (Figure 2A) and not being miRNA regulated (Figure 2B). For example, long protein length, identified with an optimal threshold of under 349 amino acids, with sequences shorter than this being 1.38 fold more likely to be associated with a gene that is not regulated by miRNAs (marked as prediction = 0). In the case of protein length, there are no missing values (i.e., all have a transcript sequence from which protein length is derived). However, the support for other features is often very limited. For example, there are only 232 proteins in the training set with the “sensory” keyword, which accounts for 2.4% of all proteins. For this selected set, the tendency to not be miRNA regulated is substantial (2.57 fold). Among the top features is membership in GPCR family 1 (associated with the olfactory receptors), which is 2.41 times less likely to be regulated (Figures 2, 3). Features directly associated with protein signaling, localization, and stability, such as post translational modification (PTM) and alternative splicing, are significant for predicting miRNA regulation. For example, proteins involved in the ubiquitination process (labelled “ubl”) are in favour of miRNA regulation by 1.26 fold, suggesting that gene regulation may involve multiple regulatory mechanisms, including dynamic tagging for degradation. Other annotations (from Gene Ontology, GO), such as subcellular components (e.g., cytosol, nucleoplasm) and interactions with RNAs (e.g., RNA binding or ribonucleoproteins), also contributed to the models’ success.

Across the different organisms, membership in various protein families was selected as a major feature, with most (but not all) members of some families sharing similar regulatory trend. Features related to amino acid sequence composition were also informative, e.g., having more than a single methionine or an especially high percentage of lysine (K). However, these characteristics are most likely a result of the underlying codon frequency (Supplementary Table S1) or indirectly a reflection of specific structural/functional families. The selection of top features is based on a combination of the Lift and RIG values to ensure maximal information gain. For example, in the model developed for *H. sapiens*, “feature contains modified residue” which specifies a modified residue for any PTM, provides the same information as the feature labelled “nonEmpty (PTM)”. This is evident when comparing all parameters, such as the enrichment for being a miRNA target relative to the prior baseline (Lift, x1.14), the number of genes in the category (support, 50%), and the RIG value (0.058). For this example, the two features are identical in their contribution to the model. Thus, only one is selected to listed among the top features.

A list of selected features and statistics is available in Supplementary Table S3. A detailed technical report that includes all features and their contributions is provided in the research GitHub. We included the model and SHAP based values (Mangalathu et al., 2020). We tested whether combined model for human and mouse is beneficial, we applied the decision tree model of XGBoost (Chen and Guestrin, 2016) trained on the combined datasets of human and mouse. Supplementary Table S3 provides the top 110 features along with their statistical analysis. The list of features according to their SHAP values is available (Supplementary Table S1, test_SHAP_features). We expect that some of these features may be confounded by the aforementioned higher-level functional properties.

Another notable set of features relates to tissue specificity. Table 3 shows the contributions of the different tissues and organs. This set of features aligns with the accepted notion that miRNA expression profiles are tissue-specific and effective at distinguishing between tissues (Ludwig et al., 2016; Rasnic et al., 2017). However, it is unknown whether some tissues are more amenable to regulation than others. Most tissue specificity features were in favor of miRNA regulation (prediction = 1), contributing to the discriminative power by ∼1.2 fold lift and a RIG of ∼0.01. Interestingly, the testis was an exception, with a 2.6 fold effect against miRNA regulation. The statistical results (Table 3) were also validated by a Chi2-based hypothesis test, complying with *p*-values <0.001. In this test, we measure the probability of observing a Chi2 deviation between the expected and observed labels of this extreme or greater (*p*-value), on the test set.

**TABLE 3:** Tissue specificity features.

Feature - tissue specificity	Dominant prediction	RIG^a^	Support (%)	Lift 1^b^	Lift 0^b^
Contains “muscle”	1	0.012	13.22	**1.19**	0.55
Contains “heart”	1	0.011	13.96	**1.18**	0.57
Contains “brain”	1	0.008	17.48	**1.14**	0.67
Contains “testis”	0	0.007	0.92	0.33	**2.57**

a Relative information gain.

b Lift indicates the effect fold for the feature on the prediction of being (Lift 1) and not being (Lift 0) miRNA, regulated.

They emphasize which class (1 - regulated, 0 - not regulated by miRNA) the feature is strongest forthe lift values refer to how much more likely the class is, given that feature. e.g., 1.2× for class 0 = class 0 is 20% more likely when that feature is true.

### Coherence among functional groups in miRNA regulation

We tested functional groups according to the UniProtKB family relations. The most significant family groups, characterized by their average regulated fraction, are shown (Figure 3). Examples with statistically significant statistics (*p* < 0.001) are listed. There are 475 protein kinases, of which 85% have been shown to be under miRNA regulation. The key signaling proteins of small GTPases (e.g., Ras, Rho) are also shown to be regulated (160 proteins, 92% are miRNA regulated). In other protein families, such as Histone H2A and H2B, none of the 17 proteins are miRNA regulated. Supplementary Table S4 lists protein families along with their prediction statistics.

### miRNA predictions in novel organisms

We examined putative predictions for less annotated model organisms such as *D. rario* (zebrafish) and *A. thaliana* (mouse-ear cress). 355 miRNA genes in *D. rario* (216 high confidence by miRBase) and 326 miRNAs in *A. thaliana* (177 high confidence by miRBase) were identified from genome and RNA-seq experiments (Zhan and Lukens, 2010). However, these organisms have a low number of experimentally validated miRNA targets, with just 187 and 70 for *D. rario* and *A. thaliana*, respectively. This is despite a comparable fraction of their genes being regulated according to computational predictions (e.g., Figure 1A). We focused our analysis on the D*. rario* with its 25,919 known proteins as retrieved from TargetScanFish. We included in the prediction scheme all non-fragmented proteins, irrespective of their annotation status. Our model, trained exclusively on annotated human genes, predicted miRNA regulation in 87% of the 22,759 genes of *D. rario* and 83% of the 30,502 genes of *A. thaliana*.

Compared to the validated shortlist of known miRNA targets, our “positive” predictions match the quantities observed in the well-studied humans and mice (∼82%). Our predicted labels also have a better concordance with TargetScan labels (Pearson correlation = 0.11) than with the small, non-represented experimentally validated sample labels (Pearson correlation = 0.027), further hinting that to a large degree, genes that are likely miRNA candidates have not yet been validated. Filtering the putative TargetScanFish to include only candidates from conserved miRNA families yields similar, improved results with a correlation of 0.12, supporting the above hypothesis. For all predictions for *D. rario* and *A. thaliana* (see Supplementary Table S1, in S2 ‘reports’ for zebrafish and thale predictions).

### Shared miRNA regulation by protein function

Our predictions suggest that miRNA regulation is prominent also in organisms lacking extensive experimental studies. The information provided to the predictor was restricted to proteomic and global functional information (see Methods). We list genes from *D. rario* that were strongly predicted to be under the miRNA regulation scheme but lack experimental evidence. Supplementary Figure S1 shows a few homologous genes of *D. rario* genes that share function with human and mouse and were also among the top predictions of miRNA regulation, with no known experimental evidence. For example, the PARK7 gene (also called DJ-1) shares 91.5% identity at the protein level between humans and mice. These are close homologs of park7 in zebrafish (with 83.1 and 81% sequence identity in human and mouse, respectively). However, applying TargetScan applied in a stringent mode for including only reliably conserved miRNAs shows that while there is no miRNA binding site in mouse transcript (ENSMUST00000030805.8), and only a single miRNA binding site in human ENST00000493678.1 transcript, TargetScan predicts 13 different miRNAs (total of 18 binding sites) in the regulation of Park7 homolog (Maillard deglycase) from zebrafish. Additional examples include the important cancer driver genes PKD2 and APC (Supplementary Figure S1).

Table 4 lists gene candidates with a high probability of being miRNA regulated in zebrafish but were not identified as such by TargetScanFish. Note that these highly predicted genes act in the nuclei during development, and are subjected to post translational modifications (PTMs). For full list of UniProtKB keynote see Supplementary Table S5. As noted, this specific model did not use any genomic or 3′-UTR length information. For most of the listed genes, zebrafish-human orthologs are associated with human disease (marked D, Table 4).

**TABLE 4:** Zebrafish miRNA predictions.

Gene name	Protein name (description)	miRNA regulation score	3′ UTR length (bp)	aHuman disease (D) UniProtKB keywords (K)
hnrnpub	Nuclear ribonucleoprotein U-like protein	91.8	1789	(D) Developmental and epileptic encephalopathy 54. (K) Phosphoprotein. Ribonucleoprotein
utp25	U3 small nucleolar RNA-associated protein 25 homolog	90.8	367	(K) Developmental protein. Nucleus. Phosphoprotein
tbxta	T-box transcription factor T-A (Brachyury protein homolog-A) (Zf-T-A)	89.8	863	(D) Sacral agenesis with vertebral anomalies. (K) Developmental protein. Transcription regulation. Wnt signaling pathway
ttn.1	Titin, tandem duplicate 1	89.6	1,001	(D) Muscular dystrophy; Dilated cardiomyopathy 1G; Hypertrophic cardiomyopathy 9. (K) ATP-binding. Immunoglobulin domain. Kinase
mib1	RING-type E3 ubiquitin transferase (EC 2.3.2.27)	89.5	1,556	(D) Left ventricular noncompaction 7 (K) Cytoskeleton. Developmental protein. Zinc-finger

a Based on ZFIN (Zebrafish Information Network) (Bradford et al., 2022).

### Integrating genomic information into a unified model

**FIGURE 4:**
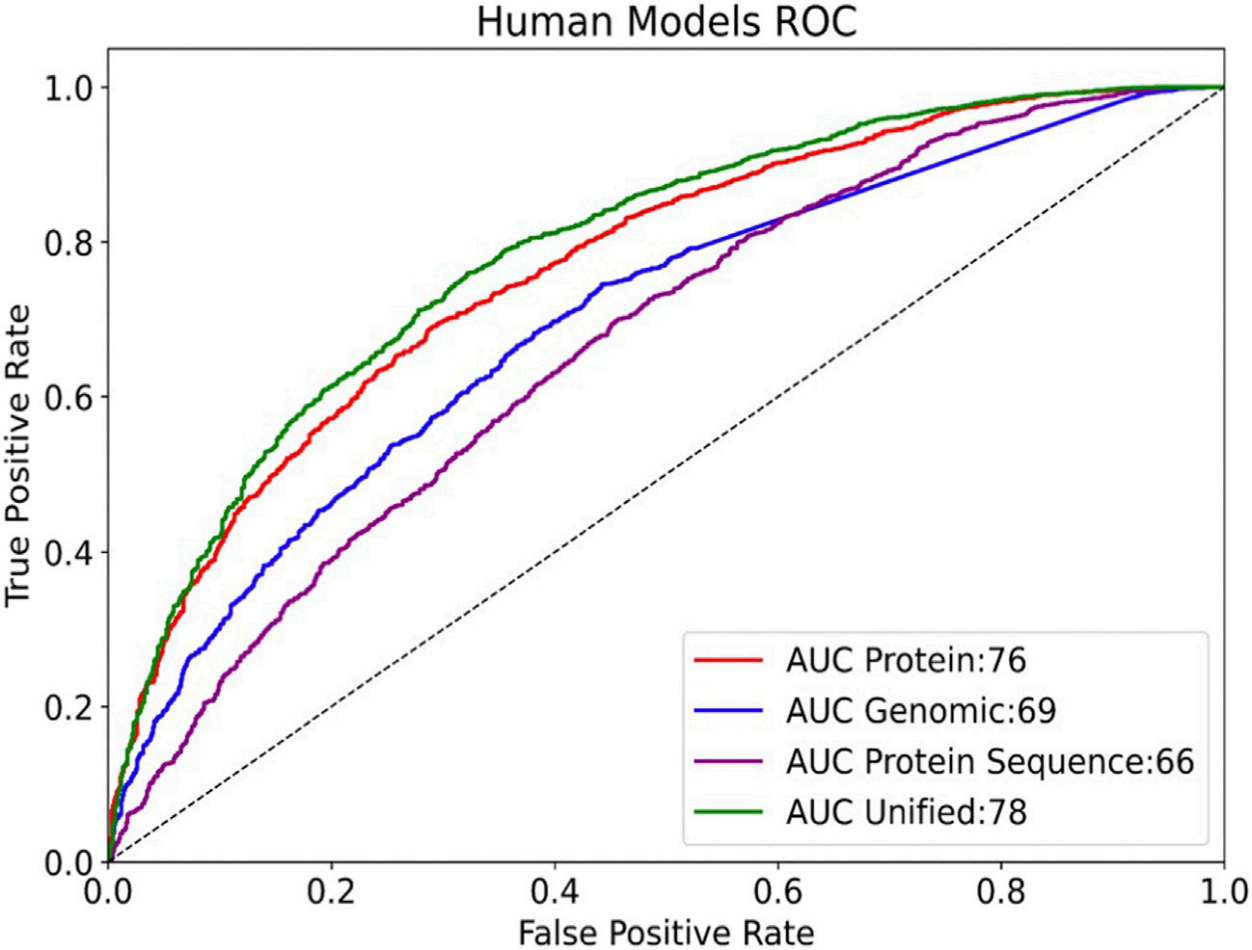
Comparing the rocAUC (Area Under the Receiver Operating Characteristic Curve) of different models on human data. Results are shown for the test set (3810 samples, 75.8% miRNA regulated). The “Protein” model includes functional annotations, keywords, primary sequences, and protein families. The “Protein sequence” model uses only protein sequence and derived features. A unified model includes all protein and genomic features.

Most computational prediction tools and algorithms for miRNA-mRNA interactions are based on sequence pairing of miRNAs to the sequence at the 3′-UTRs. We tested whether the information that is captures by the seed (6-mer) can be used to replace the information gained from the protein-based model. To this end, we added an additional set of genomic features extracted from the gene’s summary statistics (length, CDS length, nucleotide composition), the 5′-UTR and 3′-UTR length, and the number of splicing variants. We repeated the model development by adding to the training binary character n-grams of size two to four and five to six from the 3′-UTR, did not improve the model performance and was not used in the final models. Despite the observation that minimal seed (6-mer) is used as the most informative feature in determining the MBS, the performance of a model using only the genomic features was inferior to that of the protein-informed model (Figure 4). Note that our model does not consider the number of miRNA binding sites, rather it includes the 3′-UTR length (which is obviously associated with an increased probability of miRNA binding sites). The model outperforms a naive Boolean heuristic based on the existence of a 3′-UTR. The most important information at the genomic level is that a gene has a long sequence (e.g., total transcript length >540 nt) and/or a long 3′-UTR. Another highly informative feature of a gene is its chromosome position (i.e., start and end). Another unexpected observation was related to the relative location of genes on the chromosomes. Specifically, genes located towards the “start” of the chromosome were less likely to be miRNA targets. Notably, none of the extracted genomic features relate to any information on specific miRNA sequences, the presence of a match with a seed, or folding energy or other energetic stability data on pairing (e.g., n-grams complementary to a list of seeds were not included). Combining the modalities into a single, unified model outperformed any of the individual ones (Figure 4). This model reached rocAUC of 78%.

### Evolutionary related genes display regulation coherence

**FIGURE 5:**
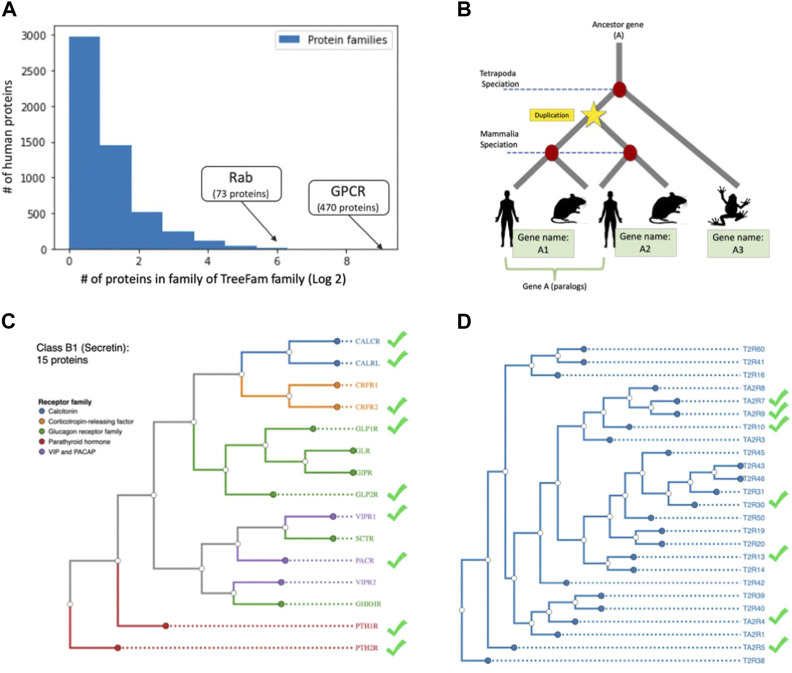
miRNA regulation of human paralogs. **(A)** The count of protein family members (log2 scale) with the number of proteins belonging to a family (*x*-axis). The largest family with 470 members of G-protein coupled receptor (GPCR) is marked. **(B)** Schematic view of homologs and paralog of ancestor gene **(A)**. The duplication event prior to specialization defines paralogs. **(C)** Phylograms of the GPCR Class B1 (Secretin) with 15 gene paralogs with nine regulated by miRNAs. The receptor groups are color coded (e.g., Glucagon receptor). **(D)** Members of GPCR of Class T (Taste 2) with 25 proteins, among them only seven proteins were predicted as miRNA regulated (marked in green symbol) the rest of the proteins were predicted as not under miRNA regulation. The source of proteins in C and D and their annotation is according to GPCRdb (Pandy-Szekeres et al., 2022). The trees are generated from whole sequence phylogenetic trees within each GPCR class using unweighted pair group method with arithmetic mean (UPGMA, 10 replicates).

We further tested whether genes that share evolutionary ancestry (i.e., orthologs) share miRNA regulation coherence. From a sequence perspective, the 3′-UTR is not under strong purifying selection across species, and in general, conservation in the 3′-UTR are minimal among orthologs. To test the cases in which miRNA regulation has been specialized, we used TreeFam as a source for family relationship groups (Schreiber et al., 2014). There are 8,819 unique TreeFam families and 5,377 protein families in this data. The question we asked is whether genes that belong to the same protein family share the same tendency towards miRNA regulation. We matched the TreeFam groups and UniProtKB-SwissProt defined protein families according to the primary gene names from the reviewed human proteome. About 90% (16,995) of the genes had a matching TreeFam gene family, and 72% had a matching protein family. We note that the remaining 10% of genes that lacked a matching TreeFam family were twice as likely to be unregulated (49% unregulated vs only 24% overall). The distribution of protein family sizes in humans is shown in Figure 5A. Note that the majority of families had only one member and thus lacked the notion of paralogs. A small number of proteins belong to multiple families. Figure 5B schematically describes the notion of paralogs.

Within TreeFam families that consisted of at least two-member genes (11,356 genes), we tested the null hypothesis, for which there is no coherence in miRNA regulation in proteins belonging to the same paralogous groups. We found that 61% of the TreeFam families, and 56% of protein families were coherent and split between the families that are all regulated (or not) by miRNAs. We further validated our hypothesis that genes belonging to the same family tend to share the same regulatory trend. The statistical test checked the greater success of assigning miRNA regulation according to the mode of each TreeFam family, for all proteins belonging to groups with at least two members. A one-sided (“greater than”) binomial test yielded a *p*-value of 1e-61. We conclude that despite poor conservation in the 3′-UTR among paralogs, families share their miRNA regulation trend, which supports our notion of functional coherence that does not necessarily rely on sequence similarity among the paralogous transcripts.

### GPCRs are rarely regulated by miRNAs

Most GPCRs are not regulated by miRNAs. We investigated whether this pattern holds true for all of the major GPCR families (470 proteins, six classes). In addition, we tested whether close paralogs of the GPCR within each class are coherent in their regulation mode of miRNA. Figure 5C shows the dendrogram of the Secretin subfamily with functional partition to receptor types (coloured coded). We confirmed that there is no direct relation between the functional relationships and the regulation mode (e.g., CRFR1/2, VIPR1/2). The same phenomenon applied to Class T (Taste 2), where seven out of 25 (28%) are known to be regulated (Figure 5D). We conclude that the selective pressure to maintain the same regulation is weakened in duplicated genes, allowing for innovation and accelerated evolution that ultimately leads to a divergence in regulation.

## Discussion

In this study, we address the question of miRNA regulation as a binary problem of prediction without considering the binding capacity, sequence specificity of each of the miRNA individually or the nature of the regulation. The information we use is mainly derived from the protein sequence and its associated annotations. For example, we showed that proteins located in the membrane or the nucleus have a higher tendency to be regulated by miRNAs (Supplementary Table S1).

Experimental evidence on miRNA regulation is scarce and fragmented. Rats and mice are rodent that are often used interchangeably as model organisms. Even in rats the number of stem-loop miRNA genes is only 40% of the number reported in mice, with only 323 miRNAs marked as high confidence. In this study, we provided a machine learning model that accurately predicts validated miRNA regulation in novel (to the model) organisms with minimal experimental results, without requiring known miRNA genes seeds (Table 4). We propose to use sequenced genomes to determine the proteome and elementary genomics properties for poorly studied organisms. The capacity of a trained model to transfer successfully across organisms is a key feature in building universal models, capable of covering all domains of life, as demonstrated in proteins language models (Ofer and Linial, 2015; Ofer et al., 2021). In addition, we anticipate that training on proteins from multiple proteomes will improve not only prediction of miRNA regulation but also related tasks (Brandes et al., 2022).

Machine learning approaches were applied in the field of miRNAs for the prediction of miRNAs from genomic information, miRNA targets of both (Singh et al., 2017; Parveen et al., 2019). All these methods are based on the properties of the molecular complementarity of miRNA and miRNA binding sites (MBS). In contrast, the strength of the automated machine learning AI model is the extensive exploration of extremely high number of features (each of our models explored millions of features) from diverse sources, both validating previous discoveries and yielding potential novel insights. We tested the predictive model on unseen data (rather than on repeated sampling of the training set). The use of an entropy-based criterion (RIG, see Methods) highlighted informative features with high discriminatory power, stability and coverage (Supplementary Figure S3). Discovery of novel sets of features that are not necessarily explainable by current knowledge, we expect to extract understanding on the biology. For example, we observed that the length of 5′-UTR, along with the length of the gene’s CDS were quite informative. Several examples confirmed that gene activation by miRNAs include binding to the 5′-UTR as shown for ribosomal proteins translation during amino acid starvation (Da Sacco and Masotti, 2012).

Another feature that contributed to the performance of the model concerns the number of alternative variants at the tail of a gene (Müller et al., 2014). In our model, the combined features of alternative polyadenylation (APA) and tissue specificity (Table 3) reflect the importance of post transcriptional regulation of the 3′-UTR as over 50% of conserved miRNAs target sites reside downstream of the proximal polyadenylation site in mammalian genes (Ren et al., 2020). It was shown that many 3′-UTR APA variants are associated with genes expressed in specific tissues and (Yang et al., 2022).

We showed that a unified model, combining proteomic and genomic modalities outperformed other models (Figure 4). In future work, we hope to experiment with models using data from multiple species simultaneously. In-depth analysis that included training on data from human and mouse indicated that the top features and the model performance remain stable and slightly improves for the species-combined model, reaching rocAUC of 80.3% (Supplementary Table S1, “reports, S2-human and mouse”). An additional benefit of proteome-based prediction in predicting miRNA in organisms lacking experimental data is with disease related orthologs. Among the top predictions in zebrafish are genes associated with human diseases including Parkinson’s disease (PARK7), cancer (APC, a known tumor suppresion gene) and kidney failure (PKD2). The shared regulation among protein families allows investigating human diseases through miRNA regulation orthologs in simpler model organisms (Chang and Mendell, 2007).

In humans, GPCRs are the largest membranous family and represent ancient duplications and further diversification. The composition of GPCR in the plasma membrane of cells are tightly regulated in health and disease. Over 400 human GPCRs (excluding hundreds that are involved in olfaction) are divided into six functional classes that are responsible for sensing smell, taste, pain, mechanical stress, vision, but also aspects of adhesion and differentiation (Pándy-Szekeres et al., 2018). We showed that GPCRs are underregulated by miRNA. Protein families such as histones are not regulated by miRNAs and their 3′-UTR is extremely short. Members of the GPCR proteins have 3′-UTR with an average length of 400-800 nucleotides, but still are mostly non-regulated by miRNAs. GPCRs act in almost every aspect of signal transduction and there are many levels of regulation that tune their activity including quantity, localization in the plasma membrane, recycling and endocytosis. We propose that translation regulation and attenuation of GPCR transcript stability by miRNAs do not contribute to the regulation of the GPCR superfamily.

We further tested the sensitivity of our proteome-based models in alternative problem formulations. About 5% of the human coding genes are marked as protein receptors, including many of the GPCR family members. We therefore trained a model only on human receptor proteins (a total of 998 proteins). Only 59% of these genes are known to be miRNA regulated (compared to 76% for the entire human proteome) The model reached a rocAUC of 84%, supporting the ability of the model to generalize, rather than merely predicting GPCRs as being unregulated. A top feature in this subpopulation was being involved in “olfaction”, covering 23% of the proteins marked as receptors.

Previous studies that sought shared properties among the miRNA regulated targets proposed that such targets are enriched in protein-protein interactions. However, large protein complexes are mostly excluded from miRNA regulation (Das et al., 2013). Inspecting the top features from the results of our unified model show that many of the miRNA regulated proteins participate in signal transduction, post translated modification, nucleic acid binding proteins (e.g., transcription factors) and cellular trafficking (e.g., small GTPase) (Figure 3). We also found that nucleosomes, ribosomes, and other stable complexes are not likely to be regulated by miRNAs. Our results argue for a design principle for miRNA regulation in which genes that participate in cell dynamics are subject to miRNA regulation, while for the function of protein complexes and structural elements (e.g. histones, ribosomes, cytoskeletal elements), the regulation by miRNA may conflict with the tight stoichiometry needed for their function.

While protein functions are under purifying selection, miRNA binding sites at the 3′-UTR are fast evolving. It has been estimated that duplicated genes in humans are twice as likely to be miRNA targets. Moreover, paralogs on average have longer 3′-UTR relative to singletons (Li et al., 2008) and the breath of regulation is greater among paralogs. Moreover, among duplicated genes that are within the same 3D topological associated domain (TAD), the coordinated expression is lower than the average non-related genes within TADs (Ibn-Salem et al., 2017). We show that protein families tend to display coherent behaviour with respect to miRNA regulation, but this coherence is not visible at the level of pairs of paralogs (Figure 5).

A number of works have proposed miRNA regulation as recent evolutionary innovation of the animal kingdom. While this could explain the differences in amount of miRNA regulated genes species, it fails at explaining the disparity between experimental and computational predictions. A more parsimonious explanation is that experimental validation is lacking, and that additional, more stable computational methods, that can also generalize across taxa, are needed to prioritize targets. We expect that a similar AI-based approach will be useful for creating a generalized model for post transcriptional regulation in living cells by ncRNAs (e.g., lncRNA, circRNA etc).

## Data Availability

The original contributions presented in the study are included in the article/Supplementary Material; further inquiries can be directed to the corresponding author. Additional data related to the paper is previously available/deposited online in a public github repository (includes code, figures, datasets, outputs)–https://github.com/LinialLab/microRNA-Protein-Regulation.

## Abbreviations

AI, artificial intelligence; CLASH, cross-linking ligation and sequencing of hybrids; CLIP, cross-linking and immunoprecipitation; GPCR, G-protein coupled receptor; miRNA, microRNA; ML, machine learning; MTI, miRNA-target interactions; ncRNA, noncoding RNA; RIG, relative information gain; rocAUC, area under the receiver operating characteristic curve; SHAP (Shapley additive explanations); TAD, topological associated domain; UPGMA, unweighted pair group method with arithmetic mean; UTR, untranslated region.

## References

[B1] Alles J. Fehlmann T. Fischer U. Backes C. Galata V. Minet M. (2019). An estimate of the total number of true human miRNAs. Nucleic Acids Res. 47 (7), 3353–3364. 10.1093/nar/gkz097 30820533 PMC6468295

[B2] Balaga O. Friedman Y. Linial M. (2012). Toward a combinatorial nature of microRNA regulation in human cells. Nucleic Acids Res. 40 (19), 9404–9416. 10.1093/nar/gks759 22904063 PMC3479204

[B3] Bradford Y. M. Van Slyke C. E. Ruzicka L. Singer A. Eagle A. Fashena D. (2022). Zebrafish information network, the knowledgebase for *Danio rerio* research. Genetics 220 (4), iyac016. 10.1093/genetics/iyac016 35166825 PMC8982015

[B4] Breuza L. Poux S. Estreicher A. Famiglietti M. L. Magrane M. Tognolli M. (2016). The UniProtKB guide to the human proteome. Database 2016, bav120. 10.1093/database/bav120 26896845 PMC4761109

[B5] Chang T.-C. Mendell J. T. (2007). microRNAs in vertebrate physiology and human disease. Annu. Rev. Genomics Hum. Genet. 8, 215–239. 10.1146/annurev.genom.8.080706.092351 17506656

[B6] Chen T. Guestrin C. (2016). Xgboost: A scalable tree boosting system. Proc. 22nd acm sigkdd Int. Conf. Knowl. Discov. data Min. 10, 785–794. 10.48550/arXiv.1603.02754

[B7] Cohen S. Dagan N. Cohen-Inger N. Ofer D. Rokach L. (2021). ICU survival prediction incorporating test-time augmentation to improve the accuracy of ensemble-based models. IEEE Access 9, 91584–91592. 10.1109/access.2021.3091622

[B8] Da Sacco L. Masotti A. (2012). Recent insights and novel bioinformatics tools to understand the role of microRNAs binding to 5'untranslated region. Int. J. Mol. Sci. 14 (1), 480–495. 10.3390/ijms14010480 23271365 PMC3565276

[B9] Das J. Chakraborty S. Podder S. Ghosh T. C. (2013). Complex-forming proteins escape the robust regulations of miRNA in human. FEBS Lett. 587 (14), 2284–2287. 10.1016/j.febslet.2013.05.062 23756149

[B10] Denzler R. Agarwal V. Stefano J. Bartel D. P. Stoffel M. (2014). Assessing the ceRNA hypothesis with quantitative measurements of miRNA and target abundance. Mol. Cell 54 (5), 766–776. 10.1016/j.molcel.2014.03.045 24793693 PMC4267251

[B11] Ding J. Zhou S. Guan J. (2010). MiRenSVM: Towards better prediction of microRNA precursors using an ensemble SVM classifier with multi-loop features. BMC Bioinforma. 11 (11), S11–S10. 10.1186/1471-2105-11-S11-S11 PMC3024864 21172046

[B12] Friedman R. C. Farh K. K.-H. Burge C. B. Bartel D. P. (2009). Most mammalian mRNAs are conserved targets of microRNAs. Genome Res. 19 (1), 92–105. 10.1101/gr.082701.108 18955434 PMC2612969

[B13] Friedman Y. Karsenty S. Linial M. (2014). Oxford: Database. miRror-Suite: decoding coordinated regulation by microRNAs. 10.1093/database/bau043 PMC4051442 24907353

[B14] Gerlach D. Kriventseva E. V. Rahman N. Vejnar C. E. Zdobnov E. M. (2009). miROrtho: computational survey of microRNA genes. Nucleic Acids Res. 37, D111–D117. Database issue). 10.1093/nar/gkn707 18927110 PMC2686488

[B15] Huang H. Y. Lin Y. C. Li J. Huang K. Y. Shrestha S. Hong H. C. (2020). miRTarBase 2020: updates to the experimentally validated microRNA-target interaction database. Nucleic Acids Res. 48 (D1), D148–D154. 10.1093/nar/gkz896 31647101 PMC7145596

[B16] Huang Z. Shi J. Gao Y. Cui C. Zhang S. Li J. (2019). HMDD v3.0: A database for experimentally supported human microRNA-disease associations. Nucleic Acids Res. 47 (1), D1013–D1017. 10.1093/nar/gky1010 30364956 PMC6323994

[B17] Ibn-Salem J. Muro E. M. Andrade-Navarro M. A. (2017). Co-regulation of paralog genes in the three-dimensional chromatin architecture. Nucleic Acids Res. 45 (1), 81–91. 10.1093/nar/gkw813 27634932 PMC5224500

[B18] Karagkouni D. Paraskevopoulou M. D. Chatzopoulos S. Vlachos I. S. Tastsoglou S. Kanellos I. (2018). DIANA-TarBase v8: A decade-long collection of experimentally supported miRNA-gene interactions. Nucleic Acids Res. 46 (D1), D239–D245. 10.1093/nar/gkx1141 29156006 PMC5753203

[B19] Kozomara A. Birgaoanu M. Griffiths-Jones S. (2019). miRBase: from microRNA sequences to function. Nucleic Acids Res. 47 (D1), D155–D162. 10.1093/nar/gky1141 30423142 PMC6323917

[B20] Lai X. Wolkenhauer O. Vera J. (2016). Understanding microRNA-mediated gene regulatory networks through mathematical modelling. Nucleic Acids Res. 44 (13), 6019–6035. 10.1093/nar/gkw550 27317695 PMC5291278

[B21] Leung A. K. Sharp P. A. (2010). MicroRNA functions in stress responses. Mol. Cell 40 (2), 205–215. 10.1016/j.molcel.2010.09.027 20965416 PMC2996264

[B22] Li J.-H. Liu S. Zhou H. Qu L.-H. Yang J.-H. (2014). starBase v2. 0: decoding miRNA-ceRNA, miRNA-ncRNA and protein–RNA interaction networks from large-scale CLIP-Seq data. Nucleic Acids Res. 42 (D1), D92–D97. 10.1093/nar/gkt1248 24297251 PMC3964941

[B23] Li J. Musso G. Zhang Z. (2008). Preferential regulation of duplicated genes by microRNAs in mammals. Genome Biol. 9 (8), R132. 10.1186/gb-2008-9-8-r132 18727826 PMC2575522

[B24] Ludwig N. Leidinger P. Becker K. Backes C. Fehlmann T. Pallasch C. (2016). Distribution of miRNA expression across human tissues. Nucleic Acids Res. 44 (8), 3865–3877. 10.1093/nar/gkw116 26921406 PMC4856985

[B25] Mackowiak S. D. (2011). Identification of novel and known miRNAs in deep‐sequencing data with miRDeep2. Curr. Protoc. Bioinforma. 36 (1), 12–15. 10.1002/0471250953.bi1210s36 22161567

[B26] Mahlab-Aviv S. Linial N. Linial M. (2019). A cell-based probabilistic approach unveils the concerted action of miRNAs. PLoS Comput. Biol. 15 (12), e1007204. 10.1371/journal.pcbi.1007204 31790387 PMC6922470

[B27] Mahlab-Aviv S. Linial N. Linial M. (2021). miRNA combinatorics and its role in cell state control-A probabilistic approach. Front. Mol. Biosci. 8, 772852. 10.3389/fmolb.2021.772852 34993232 PMC8724548

[B28] Mangalathu S. Hwang S.-H. Jeon J.-S. (2020). Failure mode and effects analysis of RC members based on machine-learning-based SHapley Additive exPlanations (SHAP) approach. Eng. Struct. 219, 110927. 10.1016/j.engstruct.2020.110927

[B29] McGeary S. E. Lin K. S. Shi C. Y. Pham T. M. Bisaria N. Kelley G. M. (2019). The biochemical basis of microRNA targeting efficacy. Science 366 (6472), eaav1741. 10.1126/science.aav1741 31806698 PMC7051167

[B51] McKinney Wes (2010). “Data structures for statistical computing in python” in Proceedings of the 9th python in science conference. McKinney: O'Reilly Media, Inc. 445, 56–61. 10.25080/Majora-92bf1922-00a

[B30] Min H. Yoon S. (2010). Got target? Computational methods for microRNA target prediction and their extension. Exp. Mol. Med. 42 (4), 233–244. 10.3858/emm.2010.42.4.032 20177143 PMC2859323

[B31] Müller S. Rycak L. Afonso-Grunz F. Winter P. Zawada A. M. Damrath E. (2014). Apadb: A database for alternative polyadenylation and microRNA regulation events. Database 2014. 10.1093/database/bau076 PMC4105710 25052703

[B32] Naamati G. Friedman Y. Balaga O. Linial M. (2012). Susceptibility of the human pathways graphs to fragmentation by small sets of microRNAs. Bioinformatics 28 (7), 983–990. 10.1093/bioinformatics/bts077 22328785

[B33] O'Brien J. Hayder H. Zayed Y. Peng C. (2018). Overview of MicroRNA biogenesis, mechanisms of actions, and circulation. Front. Endocrinol. 9, 402. 10.3389/fendo.2018.00402 PMC6085463 30123182

[B35] Ofer D. Linial M. (2015). ProFET: Feature engineering captures high-level protein functions. Bioinformatics 31 (21), 3429–3436. 10.1093/bioinformatics/btv345 26130574

[B34] Ofer D. Brandes N. Linial M. (2021). The language of proteins: NLP, machine learning & protein sequences. Comput. Struct. Biotechnol. J. 19, 1750–1758. 10.1016/j.csbj.2021.03.022 33897979 PMC8050421

[B36] Pandy-Szekeres G. Esguerra M. Hauser A. S. Caroli J. Munk C. Pilger S. (2022). The G protein database, GproteinDb. Nucleic Acids Res. 50 (D1), D518–D525. 10.1093/nar/gkab852 34570219 PMC8728128

[B37] Pándy-Szekeres G. Munk C. Tsonkov T. M. Mordalski S. Harpsøe K. Hauser A. S. (2018). GPCRdb in 2018: Adding GPCR structure models and ligands. Nucleic Acids Res. 46 (D1), D440–D446. 10.1093/nar/gkx1109 29155946 PMC5753179

[B38] Parveen A. Mustafa S. H. Yadav P. Kumar A. (2019). Applications of machine learning in miRNA discovery and target prediction. Curr. Genomics 20 (8), 537–544. 10.2174/1389202921666200106111813 32581642 PMC7290058

[B39] Quillet A. Saad C. Ferry G. Anouar Y. Vergne N. Lecroq T. (2019). Improving bioinformatics prediction of microRNA targets by ranks aggregation. Front. Genet. 10, 1330. 10.3389/fgene.2019.01330 32047509 PMC6997536

[B40] Raschka S. Mirjalili V. (2019). Python machine learning: Machine learning and deep learning with Python, scikit-learn, and TensorFlow 2. Uk: Packt Publishing Ltd.

[B41] Rasnic R. Linial N. Linial M. (2017). Enhancing identification of cancer types via lowly-expressed microRNAs. Nucleic Acids Res. 45 (9), 5048–5060. 10.1093/nar/gkx210 28379430 PMC5435932

[B42] Ren F. Zhang N. Zhang L. Miller E. Pu J. J. (2020). Alternative polyadenylation: A new frontier in post transcriptional regulation. Biomark. Res. 8 (1), 67–10. 10.1186/s40364-020-00249-6 33292571 PMC7690165

[B43] Riffo-Campos Á. L. Riquelme I. Brebi-Mieville P. (2016). Tools for sequence-based miRNA target prediction: What to choose? Int. J. Mol. Sci. 17 (12), 1987. 10.3390/ijms17121987 PMC5187787 27941681

[B44] Ritchie W. Flamant S. Rasko J. E. (2009). Predicting microRNA targets and functions: Traps for the unwary. Nat. Methods 6 (6), 397–398. 10.1038/nmeth0609-397 19478799

[B45] Romero-Cordoba S. L. Salido-Guadarrama I. Rodriguez-Dorantes M. Hidalgo-Miranda A. (2014). miRNA biogenesis: Biological impact in the development of cancer. Cancer Biol. Ther. 15 (11), 1444–1455. 10.4161/15384047.2014.955442 25482951 PMC4622859

[B46] Sayed D. Abdellatif M. (2011). MicroRNAs in development and disease. Physiol. Rev. 91 (3), 827–887. 10.1152/physrev.00006.2010 21742789

[B47] Schreiber F. Patricio M. Muffato M. Pignatelli M. Bateman A. (2014). TreeFam v9: A new website, more species and orthology-on-the-fly. Nucleic Acids Res. 42 (D1), D922–D925. 10.1093/nar/gkt1055 24194607 PMC3965059

[B48] Sethupathy P. Megraw M. Hatzigeorgiou A. G. (2006). A guide through present computational approaches for the identification of mammalian microRNA targets. Nat. Methods 3 (11), 881–886. 10.1038/nmeth954 17060911

[B49] Singh S. Benton R. G. Singh A. Singh A. (2017). Machine learning techniques in exploring microRNA gene discovery, targets, and functions. Methods Mol. Biol. 1617, 211–224. 10.1007/978-1-4939-7046-9_16 28540688

[B50] Valencia-Sanchez M. A. Liu J. Hannon G. J. Parker R. (2006). Control of translation and mRNA degradation by miRNAs and siRNAs. Genes Dev. 20 (5), 515–524. 10.1101/gad.1399806 16510870

[B52] Vishnoi A. Rani S. (2017). MiRNA biogenesis and regulation of diseases: An overview. Methods Mol. Biol. 1509, 1–10. 10.1007/978-1-4939-6524-3_1 27826912

[B53] Yang X. Tong Y. Liu G. Yuan J. Yang Y. (2022). scAPAatlas: an atlas of alternative polyadenylation across cell types in human and mouse. Nucleic Acids Res. 50 (D1), D356–D364. 10.1093/nar/gkab917 34643729 PMC8728290

[B54] Yates A. Akanni W. Amode M. R. Barrell D. Billis K. Carvalho-Silva D. (2016). Ensembl 2016., Ensembl 2016. Nucleic Acids Res. 44 (D1), D710–D716. 10.1093/nar/gkv1157 26687719 PMC4702834

[B55] Yue D. Liu H. Huang Y. (2009). Survey of computational algorithms for microRNA target prediction. Curr. Genomics 10 (7), 478–492. 10.2174/138920209789208219 20436875 PMC2808675

[B56] Zhan S. Lukens L. (2010). Identification of novel miRNAs and miRNA dependent developmental shifts of gene expression in *Arabidopsis thaliana* . PLoS One 5 (4), e10157. 10.1371/journal.pone.0010157 20405016 PMC2854152

