# Supplementary material for: Inferring microRNA regulation: A proteome perspective
Source: Front Mol Biosci. 2022 Sep 8;9:916639. doi: 10.3389/fmolb.2022.916639 (PMC9493312; doi:10.3389/fmolb.2022.916639)
Supplement: Supplementary file 3 [file DataSheet1.PDF]

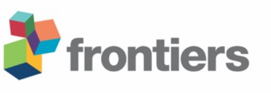

Inferring microRNA regulation: A proteome perspective

Dan Ofer, Michal Linial*

Supplementary **Figure S1.**

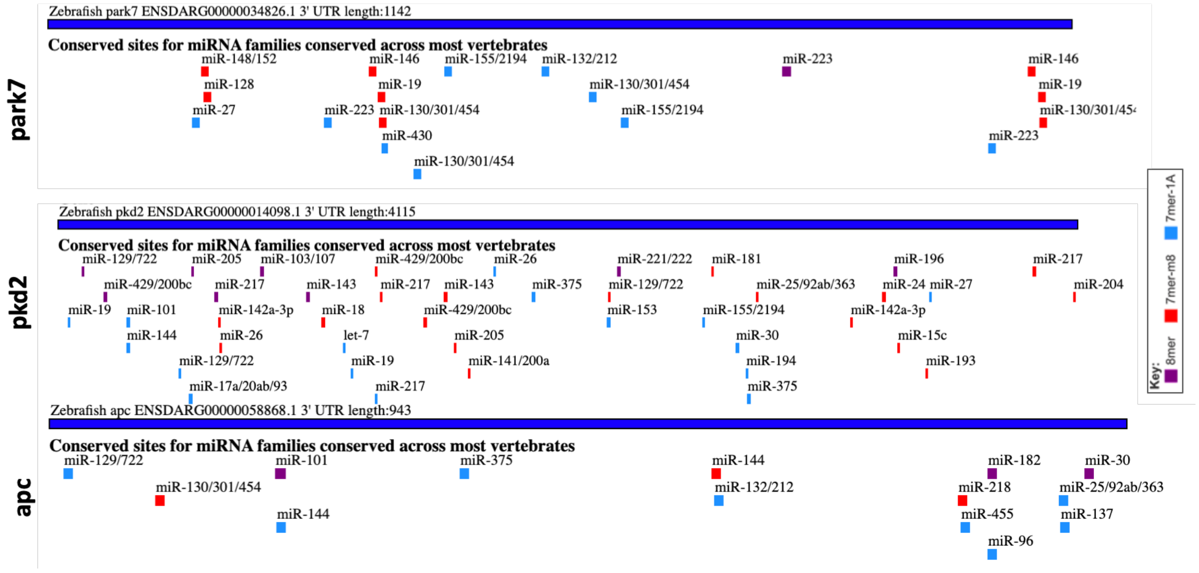

FIGURE S1. TargetScanFish viewer for three top predicting transcripts that were predicted to be under miRNA regulation by our AutoML-based model. The miRNA binding sites are shown along the 3'-UTR of zebrafish. Each binding site is colored according to the match of the seed. The colors purple, red and blue indicate a match of 8mer, 7mer-m8 and 7mer-1A, respectively. The length of the 3'-UTR are listed along with the gene name and transcript index of Ensembl.
